# Fibrinolysin production by *Alcaligenes faecalis* strain 26 isolated from environment

**Published:** 2019-08

**Authors:** Zahra Nikkhoy, Hossein Motamedi

**Affiliations:** 1 Department of Biology, Faculty of Science, Shahid Chamran University of Ahvaz, Ahvaz, Iran; 2 Biotechnology and Biological Science Research Center, Shahid Chamran University of Ahvaz, Ahvaz, Iran

**Keywords:** Fibrinolysin, Blood clot, *Alcaligenes faecalis*

## Abstract

**Background and Objectives:**

Fibrinolytic drugs are commonly used for fibrin clot lysis but due to their inappropriate side effects, as well as their high costs, using fibrinolytic enzymes has been paid attention. Bacterial sources of this enzyme are a good alternative for this purpose. The aim was fibrinolysin production through screening of fibrinolysin producing bacteria from environmental samples.

**Materials and Methods:**

Bacterial isolation was performed from different environmental samples and was screened based on sheep blood clot digestion and culture on plasma plate. The most potent isolate was optimized for different growth parameters including temperature, pH and fibrinolysin production at optimum growth conditions. The stability of produced enzyme at various temperatures and pH and treatment with MgSO_4_, NiSO_4_, SDS and EDTA was then investigated. Finally this isolate was identified based on the 16S rRNA sequencing.

**Results:**

As a result, from 79 different isolates, the most potent fibrinolysin producer was identified as *Alcaligenes faecalis* strain 26. This isolate produced 12 mm halo zone on plasma plate. Its optimum growth temperature and pH was 43°C and 7, respectively. The produced enzyme had the best stability at pH 7 and was also active up to 60°C. The fibrinolytic activity of this isolate was reduced following treatment with MgSO_4_, NiSO_4_ and also with protease inhibitors, such as SDS and EDTA.

**Conclusion:**

Based on the obtained results it can be suggested that *Alcaligenes faecalis* strain 26 has appropriate efficiency for fibrinolysin production that can be used in food industry and medicine.

## INTRODUCTION

Fibrin clot formation and lysis is a balanced event in biological system (1, 2). However, in unbalanced conditions as a result of pathophysiological disorders, fibrinolysis will be disrupted (3, 4). Fibrin precipitation in blood vessels will increase the possibility of thrombosis as well as other cardiovascular disorders such as high blood pressure, infarction, heart ischemia, environmental vascular disease, arrhythmia, brain infarction and atherosclerosis (5). Thrombosis is yet a problem in medical devices such as catheters, stents and dialysis systems. Stent thrombosis causes heart infarction and death in more than 70 percent of patients. Vascular and tissue thrombosis is occurred in 5–17 percent of dialysis patients (6). During blood coagulation, 3 events are happened: 1) following vascular damage, the external coagulation pathway is activated. The internal pathway is also activated following blood contact with activating surfaces. The factor Xa along with factor V and calcium activate the prothrombin activator. 2) Prothrombin activator catalyses the conversion of prothrombin to thrombin, and 3) fibrin, which is the main component of blood clot, is produced from fibrinogen as a result of the thrombin function. Fibrin fibers will trap platelets, erythrocytes and plasma which consequently blood clot will be produced. This clot will be stable till plasminogen activation. Tissue plasminogen activator activates plasminogen and produces plasmin, i.e., fibrinolysin which causes fibrin degradation and regeneration of damaged tissue (7, 8). With regard to this physiological fibrin degradation, fibrinolysins are used for treatment of inappropriate fibrin clots. Based on their mechanism of action, thrombolytic agents are classified in two categories: 1) plasminogen activators that act based on plasminogen activation and plasmin production, and 2) plasmin like proteins that can directly degrade fibrin clot such as earthworm lumbrokinase and fibrolase obtained from snake venom. At present, the available fibrinolysins that are used for medicinal purposes are mostly plasminogen activators such as urokinase and streptokinase which are commonly administered for emergency patients.

In spite of widespread application of these fibrinolytic agents for treatment of thrombosis, their high cost and adverse side effects such as allergies, gastrointestinal hemorrhagia, short life time, rapid inactivation and low specificity for fibrin, have restricted their application. So, researchers around the world are actively search for new fibrinolysin enzymes with high efficiency and specificity which are safe and inexpensive for medicinal purposes. Such enzymes have been found in food and non-food resources (9). From the different available sources of fibrinolysins, bacteria have been paid more attention. Because of rapid growth, low growth requirement and feasibility of their genetic engineering, these are preferred for finding new fibrinolysins with appropriate characteristics (10). The aim of this study was to find fibrinolytic bacteria from environmental samples and optimization of their growth and enzyme production properties. With regard to this fact that each ecosystem has its unique condition and so creates different habitats for bacterial colonization, screening of different environmental samples can provide a diverse source for finding new strains with desired properties. So, screening environmental samples was regarded in this study for finding fibrinolysin producing bacteria.

## MATERIALS AND METHODS

### Sampling.

The bacterial isolates used in this study were selected from bacterial collection of microbiology laboratory. These strains were isolated in previous studies and were originated from different environmental samples including agricultural soils, animal feces, plant rhizospheres, plant residue, river sediment and aquaculture pond sediments.

### Sample screening.

In order to find fibrinolysin producing bacteria, the isolates were screened through blood clot digestion and clot digestion in plasma plate methods. The bacterial isolates were cultured in nutrient broth (Merck, Germany) and incubated at 150 rpm continuous shaking (Fan Azma Gostar, Iran) at 30 or 37°C for 24 h. The broth was then centrifuged (Vision scientific, Korea) at 10000 rpm for 5 min and the supernatant was stored at 4°C. Sheep blood clots were added to phosphate buffer saline (PBS) and centrifuged at 6000 rpm for 5 min at 4°C in order to remove excess blood from clot. The obtained supernatant was mixed in 1:1 ratio with PBS and a piece of blood clot was added to this solution. This mixture was incubated at 37°C for 24 h. Simultaneously, a negative control containing distilled water instead of culture supernatant, was also prepared. The positive reaction was defined based on clot deformation and color change of the reaction solution (11). Those isolates that were able to digest clot were confirmed based on plasma plate method. Plasma plates were prepared using 1 ml human plasma, 1 ml thromboplastin D (Fisher, USA) and 4 ml of 1% agar (Merck, Germany) in sterile glass plate. Then a sterile blank disc (Padtan Teb, Iran) was saturated with the supernatant (10000 rpm, 3 min) obtained from 24 h culture of selected isolates and placed on the prepared plates. These plates were incubated at 37°C for 24 h and then the diameter of halo zone formed around each disc was measured and recorded (mm). Simultaneously, a saturated disc with non-inoculated broth was used as negative control. Based on the halo zone diameter, the most potent isolate was selected for further analysis (12, 13).

### Optimization of growth parameters.

In order to find the best growth conditions, optimization of growth temperature and pH was regarded based on one factor at time assay. The growth curve of isolate in nutrient broth was obtained at 30, 37 and 43°C. Then, the bacterial strains were cultured in pH 6, 7 and 8 and incubated at their optimum temperature obtained from previous experiments. Furthermore, the fibrinolytic activity of the selected isolate at optimum temperature and pH was evaluated based on plasma plate method.

### Enzyme properties evaluation.

For finding the cell associated or secreted nature of fibrinolysin, the broth culture of isolate was sonicated (ESM, Germany) and then the fibrinolytic activity was assessed in both sonicated and non-sonicated bacterial culture. For this purpose, bacterial culture was centrifuged (14000 rpm, 30 min) and supernatant was discarded. The precipitate was then suspended in 2 ml distilled water and sonicated during four 15 s episodes. Following centrifugation at 10000 rpm for 5 min, the supernatant was harvested and its fibrinolytic activity was assessed based on plasma plate method. The stability and function of fibrinolysin at different temperatures, pH and treatment with metal ions and protease inhibitors was also investigated. The supernatant (10000 rpm, 5 min) of 24 h bacterial culture at optimum pH and temperature was harvested and subjected to different treatments as follow and the fibrinolytic activity of treated samples was evaluated based on plasma plate method. The supernatant was separately treated for 60 min at 60, 70 and 80°C and also pH 4, 5, 6, 7 and 8. Controls were also regarded at 37°C and pH 7 (14). In order to find the effect of metal ions and protease inhibitors on fibrinolysin, the supernatant was treated with MgSO_4_ and NiSO_4_ and SDS and EDTA as protease inhibitors. For each compound, 1 mM and 5 mM concentrations were prepared in McIlvaine buffer (pH 7). Then the supernatant of bacterial culture was mixed separately with each of the prepared solutions in 1:1 (v/v) ratio and incubated at 40°C for 60 min. Following treatment, the fibrinolytic activity was evaluated in plasma plate assay and based on the differences in halo zone formation in comparison with untreated sample, the effect of metal ions and protease inhibitors on enzyme activity was concluded (14).

### Bacterial identification.

Sequencing of 16S rRNA gene was used for strain identification. For this purpose DNA was extracted using boiling method and the DNA was precipitated through cold ethanol treatment at −20°C (15). Forward (5′-CCGAATTCGTCGACAACAGAGTTTGATCCTGGCTCAG-3′) and reverse (5′-CCCGGGATCCAAGCTTACGGTTACCTTGTTACGACTT-3′) primers (16) were used in 25 μl PCR reaction containing 12.5 μl of 2× master mix (Amplicon, Denmark), 0.4 ρmol/μl of each forward and reverse primers, 1 μl from template DNA and MiliQ water. Polymerization was performed in icycler (BioRad, USA) according to the following program: denaturation (94°C, 5 min), 32 cycles each consisted of denaturation (94°C, 60 s), annealing (55°C, 40 s) and elongation (72°C, 120 s) and a final extension (72°C, 10 min). Amplification was confirmed through electrophoresis in 1% agarose containing DNA safe stain and documented by gel documentation system (Uvi tech, UK). Finally, the PCR product was sequenced (Macrogen, South Korea) and edited by Bioedit (version 7.2.6.1) and then compared with the available data in Gene bank using BLAST analysis.

## RESULTS

### Sample screening.

In this study, 79 bacterial isolates were screened for fibrinolysin production. In sheep blood clot assay, 44 isolates were able to digest the blood clot. These isolates were subjected to plasma plate assay that as a result 33 isolates were produced clear halo zone around the prepared disc. These results were confirmed fibrinolysin production by isolates. The strain 26 that was originated from Karoon river sediment showed the highest (12 mm) halo zone on plasma plate assay and hence was selected for further analysis.

### Bacterial identification.

This isolate was identified based on 16S rRNA sequencing as *Alcaligenes faecalis* strain 26.

### Optimization of growth parameters.

**Fig. 1:**
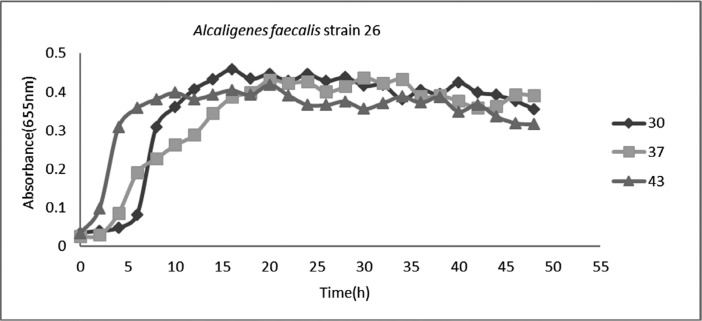
Growth curve of *Alcaligenes faecalis* strain 26 at different temperatures.

**Fig. 3:**
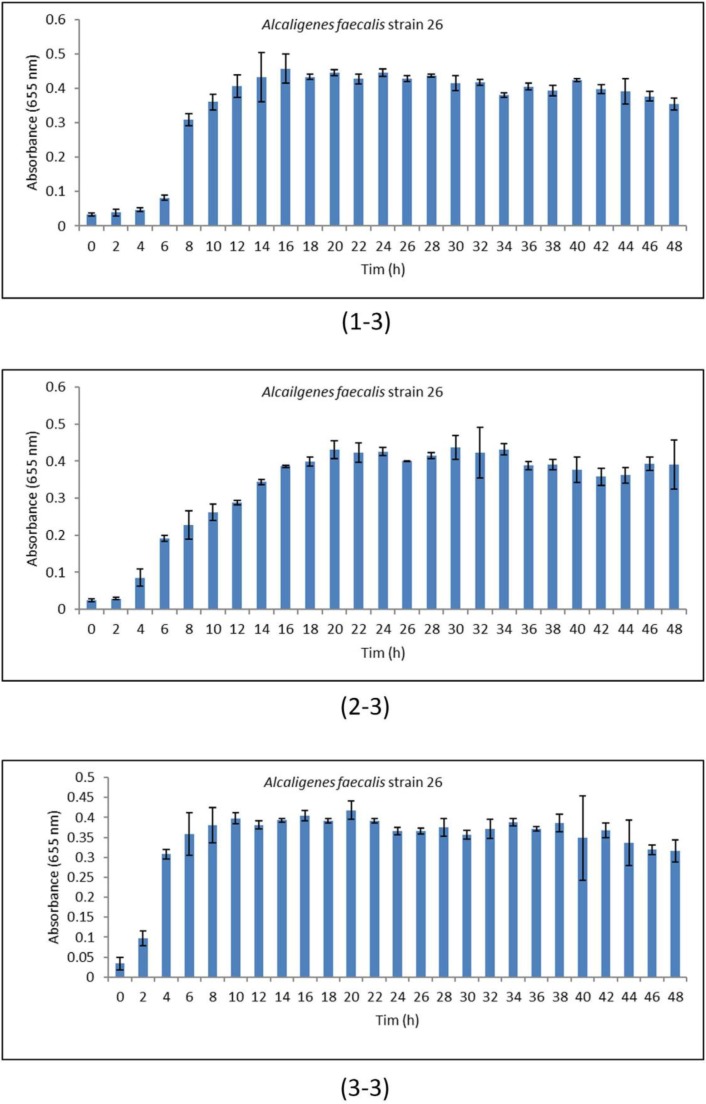
Standard deviation curve of *Alcaligenes faecalis* strain 26 at different temperature. (3-1) 30°C, (3-2) 37 °C, (3-3) 43°C

As can be found from Fig. 1, this isolate has the most growth rate at 43°C than other temperatures. So, this temperature was regarded as the optimum temperature (Figs. 1 and 3).

**Fig. 2:**
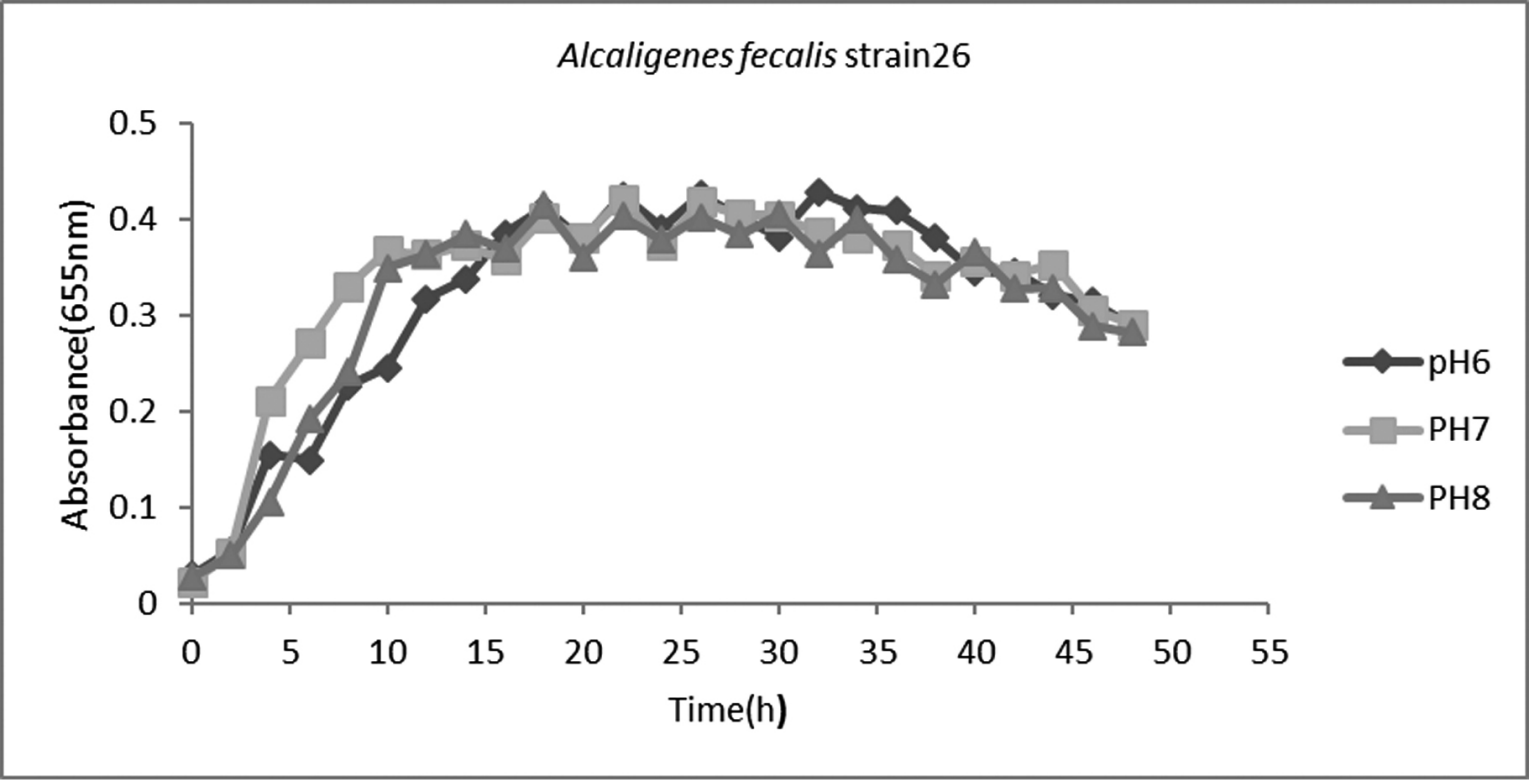
Growth curve of *Alcaligenes faecalis* strain 26 at pH 6, 7 and 8.

**Fig. 4:**
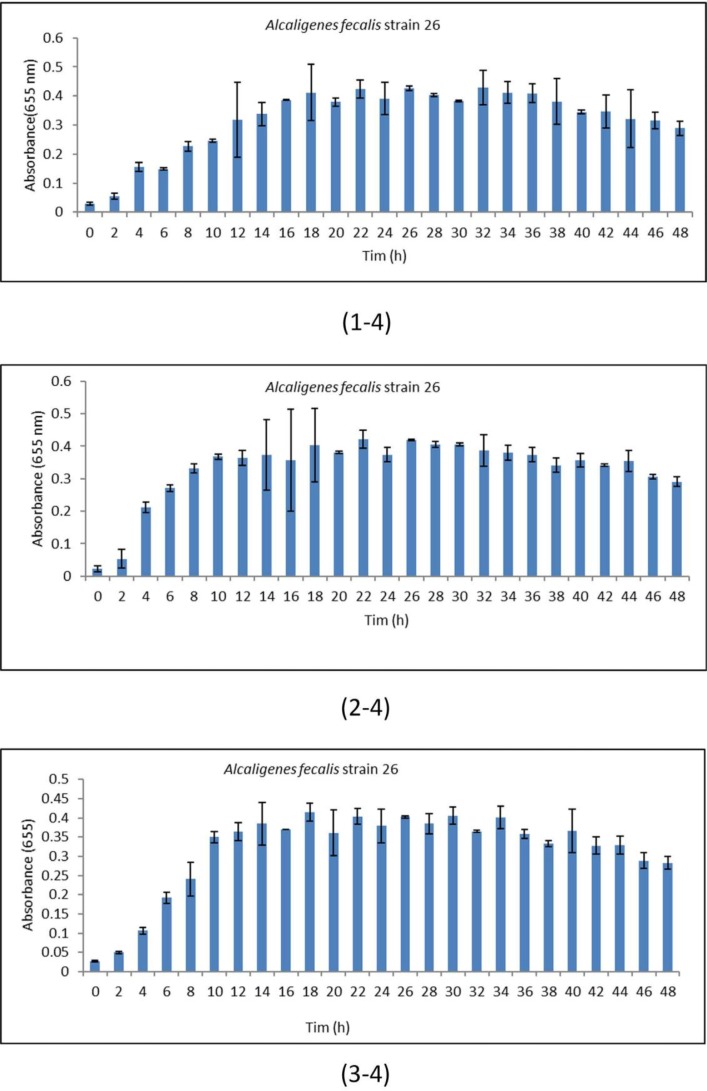
Standard deviation curve of *Alcaligenes faecalis* strain 26 at (4-1) pH 6, (4-2) 7 and (4-3) 8.

In pH optimization, it was found that this isolate has the ability to grow at all tested pH but has the best growth at neutral pH (Figs. 2 and 4).

### Enzyme properties.

In order to find that the produced fibrinolysin is totally secreted or is intracellular, sonication was performed. The results of plasma plate assay for sonicated and non-sonicated samples revealed 11.5 and 9 mm halo zone, respectively. This means that the majority of fibrinolysin is secreted and a small portion of it is intracellular. So, this is regarded as a positive point for this isolate that its product is mainly secreted and there is no need to lyse bacterial cell for enzyme extraction. The fibrinolysin of *Alcaligenes faecalis* strain 26 was stable till 60°C and pH 7. Its activity was reduced as a result of treatment with metal ions and protease inhibitors. The treated enzyme with 1 mM concentration of MgSO_4_, NiSO_4_, SDS and EDTA produced 10, 11.5, 8 and 8 mm halo zones, respectively; while at 5 mM concentration the halo zone diameter was reduced as 8, 10, 6 and 7 mm, respectively. These findings suggest that the fibrinolysin actively is affected by metal ions and protease inhibitors.

## DISCUSSION

Enzymes are important catalysts in medicine, industry, chemistry, food processing and agriculture and play central role in biochemical processes. They have notable catalytic activity that is mostly more than synthetic or organic catalysts. They also have substrate specificity, increase significantly the rate of chemical reactions and work well in aqueous solutions at mild temperature and pH. While a few other catalysts are available with all of these properties. So, today, enzymes have gained specific functions in different industries (17). From different natural sources for enzyme production, bacteria have attracted more attention because of their rapid growth, low growth requirement and the possibility of genetic engineering for optimization of their production (18). Fibrinolysin is one of the enzymes that has wide application in medicine and food industries. So, this study was conducted to achieve native bacteria able to produce and secret this enzyme. For this purpose, screening of environmental samples from Khuzestan province, southwest of Iran, was performed. This region is a tropical and semiarid habitat so bacterial inhabitants of such habitat are good strains for industrial production. Different samples as mentioned in materials and methods section were selected and their fibrinolysin production was assessed. This broad range sampling will help us to find more versatile species with fibrinolytic activity. In first screening step, sheep blood clot digestion was used. In this assay the fibrinolytic bacteria will digest the clot and hence clot deformation and color change of the clot supernatant is regarded as fibrinolysin production. However, false positive reactions may be appeared in some cases that can be due to the releasing of the trapped blood in clot that was not thoroughly extracted during clot washing. Therefore, secondary screening on plasma plate was included to confirm the potent fibrinolytic bacteria. As we can found from the results, 33 isolates from 44 isolates of primary screening were confirmed in plasma plate assay, similar results were also reported in previous studies (13). In the study of Gopal Gad et al. (2014), *Bacillus amyloliquefaciens* and *Bacillus licheniformis* were isolated from spoilt milk and soyflour, respectively, exhibited fibrinolytic activity with blood clot dissolution assay. Ho ko et al. (2004), isolated a fibrinolysin producing bacterium namely *Bacillus subtilis* QK02 based on plasma plate assay from dry soya. Latridis et al. (2015), used plasma plate assay for evaluation of the thrombolysis and fibrinolysis activity of Streptokinase enzyme.

*Alcaligenes faecalis* strain 26 that was introduced in this study was isolated from Karoon river sediment. In growth optimization it was found that this strain can grow at all tested temperatures but at 43°C has the most rapid growth and during 10 h can reach to the maximum growth at this temperature. Furthermore, pH 7 was the best pH for optimum growth. One important factor in industrial scale bacterial enzyme production is that the product be secreted from bacterial cell and not be stored intracellular. If the enzyme be secreted to extracellular environment, its purification is easy thorough centrifugation and bacterial cell precipitation. So, without need to remove biomass, enzyme production can be continued. While in cases that enzyme is intracellular, bacterial cell lysis must be done to extract enzyme. So, biomass will be missed and more time and resources must be used to reach the desired biomass. The result of plasma plate assay following bacterial sonication revealed that no significant differences are there between fibrinolytic activity of sonicated and non-sonicated bacterial cells. So, it can be concluded that the main portion of produced fibrinolysin by this strain is secreted that can be easily harvested from bacterial culture medium. Enzyme stability in different environmental conditions is of great importance in enzyme production and usage. Treatment of the fibrinolysin in this study showed that the produced enzyme is stable at a broad range of pH but have the best activity in pH 7. This property in industrial enzyme production is an advantage because due to possible changes of pH the enzyme will not be deactivated. The obtained results are in agreement with results of Jo et al. (2011), that have reported the fibrinolysin production ability of *Bacillus amyloliquefaciens* MJ 5-41, isolated from Mejo (a traditional fermented food) with best enzyme stability at pH 7 (19).

Following heat treatment of the fibrinolysin, it was found that the fibrinolysin of *Alcaligenes faecalis* strain 26 is stable till 60°C. Kim et al. (1996) have also reported that the fibrinolysin of *Bacillus* sp. strain CK11-4 is stable till 65°C (20). This finding is also in agreement with our data.

The effect of metal ions and protease inhibitors on enzyme stability was investigated because such agents may be affect the enzyme activity during their application. The results showed that NiSO_4_ and MgSO_4_ cause increase and decrease in fibrinolysin activity, respectively and both SDS and EDTA reduced the enzyme activity. Lee et al. (2001), found that Ni^2+^ at 5 μm concentration increased 20–40% of fibrinolysin activity of *Bacillus* sp. Strain K DO-13. EDTA was significantly inhibited the enzyme activity which was parallel to its concentration and cobalt addition was restored the enzyme activity. So their fibrinolysin was a cobalt dependent metaloprotease (14).

## CONCLUSION

The results of this study suggest that screening of different environmental samples can provide an opportunity for finding new strains with fibrinolytic potential. The introduced *Alcaligenes faecalis* strain 26 in this study with regard to its enzyme stability at 60°C is a good strain for enzyme production because there isn’t any need to cold chain for enzyme storage and transportation. The product of this strain has advantages that can be used for medicinal applications and food processing.
